# (*E*)-3-(4-Bromo­phen­yl)-1-(3,4-dichloro­phen­yl)prop-2-en-1-one

**DOI:** 10.1107/S1600536809008162

**Published:** 2009-03-25

**Authors:** Rajni Kant, Kamni, B. Narayana, K. Veena, H. S. Yathirajan

**Affiliations:** a Department of Physics, University of Jammu, Jammu Tawi 180 006, India; b School of Applied Physics and Mathematics, Shri Mata Vaishno Devi University, Jammu 182 121, India; c Department of Studies in Chemistry, Mangalore University, Mangalagangotri 574 199, India; d Department of Studies in Chemistry, University of Mysore, Manasagangotri 576 006, India

## Abstract

The mol­ecule of the title compound, C_15_H_9_BrCl_2_O, is shown to be the *E* isomer, with the 3,4-dichloro­benzoyl and *p*-bromo­phenyl substituents in *trans* positions with respect to the chalcone olefin bond. The mol­ecule is non-planar, the two aromatic rings forming a dihedral angle of 49.58 (1)°.

## Related literature

For related literature on chalcones, see: Dhar (1981 ▶); Di Carlo *et al.* (1999 ▶); Dimmock *et al.* (1999 ▶); Go *et al.* (2005 ▶); Sarojini *et al.* (2006 ▶). For related structures, see: Li *et al.* (2007 ▶, 2008 ▶); Wang *et al.* (2007 ▶); Tiang *et al.* (2007 ▶); Teh *et al.* (2006 ▶); Patil *et al.* (2006 ▶); Butcher *et al.* (2007 ▶).
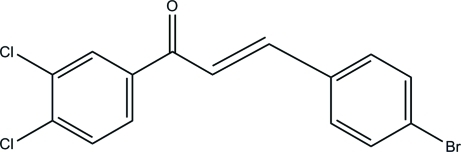


## Experimental

### 

#### Crystal data

C_15_H_9_BrCl_2_O
*M* *_r_* = 356.05
Triclinic, 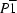

*a* = 5.9370 (5) Å
*b* = 7.7365 (6) Å
*c* = 14.8254 (11) Å
α = 81.347 (6)°
β = 88.182 (6)°
γ = 88.315 (6)°
*V* = 672.66 (9) Å^3^
*Z* = 2
Mo *K*α radiation
μ = 3.44 mm^−1^
*T* = 293 K
0.30 × 0.24 × 0.18 mm

#### Data collection

Oxford Diffraction Xcalibur diffractometer
Absorption correction: multi-scan (*SADABS*; Sheldrick, 2004 ▶) *T*_min_ = 0.383, *T*_max_ = 0.538
7411 measured reflections
3671 independent reflections
2762 reflections with *I* > 2σ(*I*)
*R*_int_ = 0.024

#### Refinement

*R*[*F* ^2^ > 2σ(*F* ^2^)] = 0.048
*wR*(*F* ^2^) = 0.100
*S* = 1.14
3671 reflections
209 parameters
All H-atom parameters refined
Δρ_max_ = 0.61 e Å^−3^
Δρ_min_ = −0.50 e Å^−3^

### 

Data collection: *CrysAlis Pro* (Oxford Diffraction, 2007 ▶); cell refinement: *CrysAlis Pro*; data reduction: *CrysAlis RED*; program(s) used to solve structure: *SHELXS86* (Sheldrick, 2008 ▶); program(s) used to refine structure: *SHELXL97* (Sheldrick, 2008 ▶); molecular graphics: *ORTEP-3 for Windows* (Farrugia, 1997 ▶); software used to prepare material for publication: *WinGX* (Farrugia, 1999 ▶).

## Supplementary Material

Crystal structure: contains datablocks global, I. DOI: 10.1107/S1600536809008162/ya2083sup1.cif

Structure factors: contains datablocks I. DOI: 10.1107/S1600536809008162/ya2083Isup2.hkl

Additional supplementary materials:  crystallographic information; 3D view; checkCIF report

## Figures and Tables

**Fig. 1.**
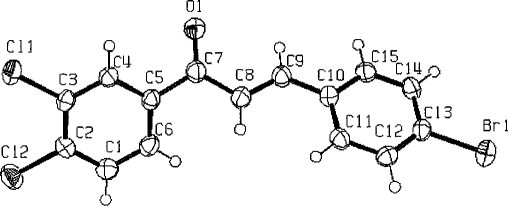
Molecular structure of the title compound; thermal displacement ellipsoids are drawn at the 50% probability level.

## supplementary crystallographic information

### Comment

1,3-Diaryl-2-propen-1-ones, also known as chalcones, belong to the flavonoid family. The radical quenching properties of the phenolic groups present in many chalcones have raised interest in using the chalcone rich plant extracts as drugs or food preservatives (Dhar, 1981). Chalcones have also been reported to possess many useful properties, including anti-inflammatory, antimicrobial, antifungal, antioxidant, cytotoxic, antitumor and anticancer activities (Dimmock *et al.*, 1999; Go *et al.*, 2005). They are also finding application as organic nonlinear optical materials (Sarojini *et al.*, 2006).

Owing to the general importance of these flavanoid analogues we report herein the synthesis and crystal structure of a new chalcone, (E)-3-(4-bromophenyl)-1-(3,4-dichlorophenyl)prop-2-en-1-one.

In the molecule of the title compound (Fig.1) the dichlorobenzoyl and p-bromophenyl substituents are in trans positions with respect to the C8=C9 double bond. The meolecule is non-planar; the dihedral angle formed by the aromatic rings C1-C6 and C10-C15 is equal to 49.58 (1)°.

### Experimental

5 ml of 50% KOH was added to a mixture of 3,4-dichloroacetophenone (0.945 g, 0.005 mol) and 4-bromobenzaldehyde (0.92 g, 0.005 mol) in 25 ml of ethanol. The mixture was then stirred for an hour at room temperature and the precipitate was collected by filtration and purified by recrystallization from ethanol (m.p. 398-402 K; yield 74%). The single crystals were grown by slow evaporation from ethyl acetate. Analytical data: Found (Cald), %: C 50.58 (50.60); H 2.51 (2.55).

### Refinement

All H atoms were located in the difference Fourier map and refined isotropically. The C—H distances are in the range of 0.90-0.96Å.

### Crystal data

**Table:**

C_15_H_9_BrCl_2_O	*Z* = 2
*M**_r_* = 356.05	*F*(000) = 352
Triclinic, *P*1	*D*_x_ = 1.758 Mg m^−^^3^
Hall symbol: -P 1	Mo *K*α radiation, λ = 0.71073 Å
*a* = 5.9370 (5) Å	Cell parameters from 2762 reflections
*b* = 7.7365 (6) Å	θ = 3.2–30.3°
*c* = 14.8254 (11) Å	µ = 3.44 mm^−^^1^
α = 81.347 (6)°	*T* = 293 K
β = 88.182 (6)°	Rectangular, pale yellow
γ = 88.315 (6)°	0.30 × 0.24 × 0.18 mm
*V* = 672.66 (9) Å^3^	

### Data collection

**Table:**

Oxford Diffraction Xcalibur diffractometer	3671 independent reflections
Radiation source: fine-focus sealed tube	2762 reflections with *I* > 2σ(*I*)
graphite	*R*_int_ = 0.024
ω–2θ scans	θ_max_ = 30.3°, θ_min_ = 3.2°
Absorption correction: multi-scan (*SADABS*; Sheldrick, 2004)	*h* = −8→8
*T*_min_ = 0.383, *T*_max_ = 0.538	*k* = −10→10
7411 measured reflections	*l* = −20→20

### Refinement

**Table:**

Refinement on *F*^2^	All H-atom parameters refined
Least-squares matrix: full	*w* = 1/[σ^2^(*F*_o_^2^) + (0.0232*P*)^2^ + 0.974*P*] where *P* = (*F*_o_^2^ + 2*F*_c_^2^)/3
*R*[*F*^2^ > 2σ(*F*^2^)] = 0.048	(Δ/σ)_max_ = 0.002
*wR*(*F*^2^) = 0.100	Δρ_max_ = 0.61 e Å^−^^3^
*S* = 1.14	Δρ_min_ = −0.50 e Å^−^^3^
3671 reflections	Extinction correction: *SHELXL97* (Sheldrick, 2008), Fc^*^=kFc[1+0.001xFc^2^λ^3^/sin(2θ)]^-1/4^
209 parameters	Extinction coefficient: 0.0163 (13)
0 restraints	

### Special details

**Table:**

Geometry. All esds (except the esd in the dihedral angle between two l.s. planes) are estimated using the full covariance matrix. The cell esds are taken into account individually in the estimation of esds in distances, angles and torsion angles; correlations between esds in cell parameters are only used when they are defined by crystal symmetry. An approximate (isotropic) treatment of cell esds is used for estimating esds involving l.s. planes.

### Fractional atomic coordinates and isotropic or equivalent isotropic displacement parameters (Å^2^)

**Table:**

	*x*	*y*	*z*	*U*_iso_*/*U*_eq_	
H4	−0.733 (6)	−0.335 (4)	0.696 (2)	0.029 (9)*	
H15	−0.435 (6)	−0.108 (4)	0.215 (2)	0.032 (9)*	
H14	−0.208 (6)	0.023 (5)	0.096 (2)	0.038 (9)*	
H1	−0.040 (6)	−0.628 (5)	0.737 (2)	0.039 (9)*	
H12	0.256 (6)	0.100 (5)	0.275 (2)	0.044 (10)*	
H6	−0.128 (6)	−0.465 (5)	0.599 (3)	0.047 (11)*	
H9	−0.520 (6)	−0.192 (5)	0.370 (2)	0.041 (10)*	
H11	0.024 (6)	−0.027 (5)	0.393 (3)	0.041 (10)*	
H8	−0.193 (6)	−0.212 (5)	0.496 (2)	0.045 (10)*	
Br1	0.22801 (7)	0.18580 (5)	0.07919 (2)	0.04765 (14)	
Cl1	−0.73344 (15)	−0.48572 (14)	0.87746 (6)	0.0488 (2)	
Cl2	−0.26813 (16)	−0.68518 (13)	0.90475 (6)	0.0491 (2)	
C12	0.1124 (6)	0.0635 (5)	0.2638 (2)	0.0365 (7)	
C13	0.0417 (5)	0.0773 (4)	0.1753 (2)	0.0339 (7)	
C4	−0.5964 (6)	−0.3937 (4)	0.7036 (2)	0.0325 (7)	
C3	−0.5425 (5)	−0.4813 (4)	0.7880 (2)	0.0308 (6)	
C10	−0.2309 (5)	−0.0822 (4)	0.3167 (2)	0.0314 (7)	
C6	−0.2335 (6)	−0.4685 (5)	0.6444 (2)	0.0377 (8)	
C11	−0.0236 (6)	−0.0170 (5)	0.3342 (2)	0.0354 (7)	
C5	−0.4425 (5)	−0.3860 (4)	0.6308 (2)	0.0321 (7)	
C2	−0.3345 (5)	−0.5675 (4)	0.8006 (2)	0.0316 (6)	
C9	−0.3824 (6)	−0.1641 (5)	0.3887 (2)	0.0358 (7)	
C15	−0.2963 (6)	−0.0639 (5)	0.2264 (2)	0.0350 (7)	
O1	−0.7120 (4)	−0.2757 (4)	0.52258 (17)	0.0547 (7)	
C1	−0.1805 (6)	−0.5621 (5)	0.7283 (2)	0.0385 (8)	
C7	−0.5123 (6)	−0.2916 (5)	0.5405 (2)	0.0374 (7)	
C14	−0.1609 (6)	0.0134 (5)	0.1552 (2)	0.0375 (8)	
C8	−0.3364 (6)	−0.2186 (5)	0.4749 (2)	0.0392 (8)	

### Atomic displacement parameters (Å^2^)

**Table:**

	*U*^11^	*U*^22^	*U*^33^	*U*^12^	*U*^13^	*U*^23^
Br1	0.0529 (2)	0.0481 (2)	0.0392 (2)	−0.00965 (17)	0.01013 (15)	0.00191 (15)
Cl1	0.0466 (5)	0.0615 (6)	0.0345 (4)	0.0011 (4)	0.0124 (3)	0.0022 (4)
Cl2	0.0572 (5)	0.0459 (5)	0.0403 (5)	0.0040 (4)	−0.0066 (4)	0.0064 (4)
C12	0.0330 (17)	0.0381 (19)	0.0386 (17)	−0.0066 (15)	−0.0010 (13)	−0.0049 (14)
C13	0.0387 (17)	0.0287 (17)	0.0320 (15)	0.0015 (14)	0.0051 (13)	0.0008 (12)
C4	0.0310 (16)	0.0332 (18)	0.0330 (16)	−0.0052 (14)	0.0002 (12)	−0.0027 (13)
C3	0.0339 (15)	0.0287 (16)	0.0297 (15)	−0.0068 (13)	0.0047 (12)	−0.0038 (12)
C10	0.0366 (16)	0.0291 (17)	0.0284 (15)	−0.0029 (13)	−0.0002 (12)	−0.0039 (12)
C6	0.0348 (17)	0.048 (2)	0.0325 (16)	−0.0062 (15)	0.0045 (13)	−0.0113 (14)
C11	0.0384 (17)	0.0394 (19)	0.0281 (15)	−0.0022 (15)	−0.0038 (13)	−0.0037 (13)
C5	0.0327 (15)	0.0360 (18)	0.0282 (15)	−0.0085 (14)	0.0007 (12)	−0.0052 (13)
C2	0.0393 (17)	0.0258 (16)	0.0303 (15)	−0.0007 (13)	−0.0054 (12)	−0.0055 (12)
C9	0.0367 (17)	0.0372 (19)	0.0332 (16)	−0.0062 (15)	−0.0016 (13)	−0.0034 (13)
C15	0.0344 (17)	0.0365 (19)	0.0343 (16)	−0.0055 (14)	−0.0047 (13)	−0.0040 (14)
O1	0.0375 (13)	0.085 (2)	0.0372 (13)	−0.0068 (14)	−0.0018 (10)	0.0060 (13)
C1	0.0371 (18)	0.039 (2)	0.0410 (18)	0.0011 (15)	−0.0018 (14)	−0.0100 (15)
C7	0.0401 (18)	0.042 (2)	0.0299 (16)	−0.0066 (15)	0.0000 (13)	−0.0042 (14)
C14	0.0461 (19)	0.040 (2)	0.0259 (15)	−0.0029 (16)	−0.0044 (13)	−0.0016 (13)
C8	0.0358 (17)	0.048 (2)	0.0328 (16)	−0.0096 (16)	−0.0014 (13)	−0.0006 (14)

### Geometric parameters (Å, °)

**Table:**

Br1—C13	1.885 (3)	C6—C5	1.385 (5)
Cl1—C3	1.714 (3)	C6—H6	0.90 (4)
Cl2—C2	1.722 (3)	C11—H11	0.92 (4)
C12—C13	1.379 (5)	C5—C7	1.491 (4)
C12—C11	1.381 (4)	C2—C1	1.382 (5)
C12—H12	0.93 (4)	C9—C8	1.319 (5)
C13—C14	1.372 (5)	C9—H9	0.91 (4)
C4—C3	1.373 (4)	C15—C14	1.379 (4)
C4—C5	1.387 (4)	C15—H15	0.93 (3)
C4—H4	0.92 (3)	O1—C7	1.222 (4)
C3—C2	1.391 (4)	C1—H1	0.96 (4)
C10—C11	1.390 (5)	C7—C8	1.470 (4)
C10—C15	1.392 (4)	C14—H14	0.92 (4)
C10—C9	1.455 (4)	C8—H8	0.92 (4)
C6—C1	1.383 (5)		
			
C13—C12—C11	119.3 (3)	C4—C5—C7	118.1 (3)
C13—C12—H12	119 (2)	C1—C2—C3	119.9 (3)
C11—C12—H12	121 (2)	C1—C2—Cl2	119.4 (3)
C14—C13—C12	121.6 (3)	C3—C2—Cl2	120.7 (2)
C14—C13—Br1	119.0 (2)	C8—C9—C10	127.7 (3)
C12—C13—Br1	119.3 (2)	C8—C9—H9	116 (2)
C3—C4—C5	120.6 (3)	C10—C9—H9	116 (2)
C3—C4—H4	120 (2)	C14—C15—C10	122.0 (3)
C5—C4—H4	120 (2)	C14—C15—H15	121 (2)
C4—C3—C2	120.0 (3)	C10—C15—H15	117 (2)
C4—C3—Cl1	119.8 (2)	C2—C1—C6	119.6 (3)
C2—C3—Cl1	120.2 (2)	C2—C1—H1	119 (2)
C11—C10—C15	117.9 (3)	C6—C1—H1	121 (2)
C11—C10—C9	122.8 (3)	O1—C7—C8	121.5 (3)
C15—C10—C9	119.3 (3)	O1—C7—C5	120.0 (3)
C1—C6—C5	120.8 (3)	C8—C7—C5	118.5 (3)
C1—C6—H6	117 (2)	C13—C14—C15	118.3 (3)
C5—C6—H6	122 (2)	C13—C14—H14	122 (2)
C12—C11—C10	120.8 (3)	C15—C14—H14	120 (2)
C12—C11—H11	119 (2)	C9—C8—C7	121.0 (3)
C10—C11—H11	120 (2)	C9—C8—H8	121 (2)
C6—C5—C4	119.1 (3)	C7—C8—H8	118 (2)
C6—C5—C7	122.9 (3)		

## References

[bb1] Butcher, R. J., Jasinski, J. P., Yathirajan, H. S., Narayana, B. & Mayekar, A. N. (2007). *Acta Cryst.* E**63**, o4253–o4254.

[bb2] Dhar, D. N. (1981). *The Chemistry of Chalcones and Related Compounds* New York: John Wiley.

[bb3] Di Carlo, G., Mascolo, N., Izzo, A. A. & Capasso, F. (1999). *Life Sci.* **65**, 337–353. 10.1016/s0024-3205(99)00120-4 10421421

[bb4] Dimmock, J. R., Elias, D. W., Beazely, M. A. & Kandepu, N. M. (1999). *Curr. Med. Chem.* **6**, 1125–1149. 10519918

[bb5] Farrugia, L. J. (1997). *J. Appl. Cryst.* **30**, 565.

[bb6] Farrugia, L. J. (1999). *J. Appl. Cryst.* **32**, 837–838.

[bb7] Go, M. L., Wu, X. & Liu, X. L. (2005). *Curr. Med. Chem.* **12**, 483–499.

[bb8] Li, H., Sarojini, B. K., Raj, C. G. D., Madhu, L. N. & Yathirajan, H. S. (2008). *Acta Cryst.* E**64**, o2238. 10.1107/S1600536808034867 PMC2959749 21581092

[bb9] Li, T.-D., Tian, N.-N., Bi, S. & Wan, J. (2007). *Acta Cryst.* E**63**, o3063.

[bb10] Oxford Diffraction (2007). *CrysAlis Pro* and *CrysAlis RED* Oxford Diffraction Ltd, Abingdon, England.

[bb11] Patil, P. S., Teh, J. B.-J., Fun, H.-K., Razak, I. A. & Dharmaprakash, S. M. (2006). *Acta Cryst.* E**62**, o1710–o1712.

[bb12] Sarojini, B. K., Narayana, B., Ashalatha, B. V., Indira, J. & Lobo, K. G. (2006). *J. Cryst. Growth*, **295**, 54–59.

[bb13] Sheldrick, G. M. (2004). *SADABS* University of Göttingen, Germany.

[bb14] Sheldrick, G. M. (2008). *Acta Cryst.* A**64**, 112–122. 10.1107/S0108767307043930 18156677

[bb15] Teh, J. B.-J., Patil, P. S., Fun, H.-K., Razak, I. A. & Dharmaprakash, S. M. (2006). *Acta Cryst.* E**62**, o4380–o4381.

[bb16] Tian, N.-N., Bi, S., Xu, L.-L. & Wan, J. (2007). *Acta Cryst.* E**63**, o3602.

[bb17] Wang, C.-Y., Xia, P., Han, Z.-P., Shen, R.-M. & Cui, N. (2007). *Acta Cryst.* E**63**, o1238–o1239.

